# Post-tubercular bronchopleural fistula with tension hydropneumothorax: a rare case and its management and diagnostic challenges in a low-income country: a case report

**DOI:** 10.1186/s13256-026-06408-x

**Published:** 2026-07-29

**Authors:** Amanuel Dagabas Wakoya, Ayto Addisu Negash, Fitsum Negusse Assefa

**Affiliations:** 1 Department of Emergency and Critical Care Medicine, Haramaya University, Harar, Ethiopia; 2 Department of Emergency and Critical Care Medicine, St. Paul’s Hospital Millennium Medical College, Addis Ababa, Ethiopia; 3 Department of Internal Medicine, St. Paul’s Hospital Millennium Medical College, Addis Ababa, Ethiopia; 4 Department of Intensive care Medicine, St. Paul’s Hospital Millennium Medical College, Addis Ababa, Ethiopia

**Keywords:** Tuberculosis, Bronchopleural fistula, Tension hydropneumothorax, Case report

## Abstract

**Background:**

A bronchopleural fistula (BPF) accompanied by tension hydropneumothorax is a rare but potentially life-threatening complication of tuberculosis, posing significant diagnostic and treatment challenges. Effective management requires a multidisciplinary approach, particularly when there are additional concurrent infections involved.

**Case presentation:**

A 28-year-old Black Ethiopian male with no significant past medical history presented to the emergency department with a 1-week exacerbation of shortness of breath, alongside a 2-month history of productive cough, weight loss, and night sweats. He was diagnosed with tuberculosis via GeneXpert testing and subsequently bronchopleural fistula complicated by hydropneumothorax with imaging. Management consisted of chest tube drainage, intravenous antibiotic therapy, and standard first-line antitubercular treatment. The patient showed significant improvement and was discharged in stable condition.

**Conclusion:**

This case highlights a rare complication of cavitary tuberculosis and demonstrates that bronchopleural fistula can be successfully managed with nonsurgical approaches, resulting in clinical recovery and lung re-expansion, while also providing insights into diagnostic and management strategies.

## Introduction

Bronchopleural fistula (BPF) refers to an abnormal connection between the bronchial tree and the pleural space [1]. The causes of BPF can include surgical removal of lung tissue, necrosis of lung tissue due to infection, trauma, and lung cancers. Tuberculous bronchopleural fistula is a rare complication of pulmonary tuberculosis, with presentation ranging from patients who are asymptomatic with incidental findings on imaging to those who present with acute tension pneumothorax [2]. In rare instances, complications from bronchopleural fistula (BPF) can lead to contamination of the pleural space, resulting in inflammation and the simultaneous presence of fluid and air. This can cause conditions that range from hydropneumothorax to empyema [1]. Although hydropneumothorax has been the subject of considerable research, instances of its occurrence alongside tuberculosis are frequently underreported in the existing literature [3]. In tuberculosis, the condition typically starts with a focus of infection, which can progress to form a cavity that eventually ruptures, releasing air and fluid into the pleural cavity [3]. We report a rare case of left-sided post-tuberculous BPF with tension hydropneumothorax, accompanied by contralateral cavitary pulmonary lesions, successfully managed with antitubercular therapy, antibiotics and chest tube drainage. This case adds to the limited literature on bilateral TB-related pulmonary sequelae and highlights the importance of tailored conservative management in select cases.

## Case presentation

We report the case of a 28-year-old Black Ethiopian male patient who presented with a complaint of a productive cough of 2 months’ duration. The cough was associated with progressive loss of appetite, significant but unquantified weight loss, and drenching night sweats. In the week prior to presentation, the cough worsened and was accompanied by shortness of breath, low-grade fever, and easy fatigability. These symptoms prompted him to seek care at our institution. He had no history of previously diagnosed chronic medical illnesses such as diabetes mellitus, hypertension, or cardiac disease. There was no history of contact with a person diagnosed with tuberculosis or anyone with chronic cough. He denied any history of smoking or alcohol consumption.

At the initial emergency department visit, he appeared acutely on chronically ill and was in respiratory distress. His vital signs were: blood pressure 90/60 mmHg, pulse rate 104 beats per minute, respiratory rate 32 breaths per minute, temperature 37.6 °C, and oxygen saturation 82% on room air. He was able to maintain oxygen saturation above 90% with 10 L of oxygen via face mask. His BMI was calculated at 17.5 kg/m^2^. On physical examination, he had pale conjunctiva, nonicteric sclera, and prominent zygomatic bones. Chest examination revealed tachypnea with use of accessory muscles, absent air entry in the lower two-thirds of the left posterior chest, and coarse crepitations in the upper third of the right chest. Musculoskeletal examination showed an emaciated appearance. His GCS was 15/15.

Based on these findings, a preliminary diagnosis of severe pneumonia and disseminated tuberculosis was considered. He was started on 10 L/min oxygen via face mask and placed under continuous monitoring. One liter of normal saline was administered as a bolus. After sending a blood culture, broad-spectrum antibiotics were initiated: cefepime 2 g IV every 8 h and vancomycin 1 g IV every 12 h. His blood pressure improved to 100/68 mmHg following the fluid bolus. Basic laboratory tests and imaging were requested (see Table 1).

**Table 1:** Laboratory result summary

Parameter	Reference range	Jan 19/2025	Jan 23/2025	Jan 25/2025	Jan 26/2025
WBC (× 10^3^/µL)	3.9–10.1	32.8	12.96	10.72	9.18
Neutrophils (%)	40.4–74.6	95.1	86.9	88.9	81.9
Lymphocytes (%)	17.8–61.5	2.8	8.61	7.0	12.7
Hemoglobin (g/dL)	12.7–17.1	10.4	8.5	9.1	9.7
Hematocrit (%)	34.4–48.3	33.4	26.3	27.9	31.7
MCV (fL)	74.3–98.3	72.1	67.9	68.6	71.8
RDW (%)	11.7–15.3	17.8	18.0	18.0	17.8
Platelets (× 10^3^/µL)	100–300	399	274	310	323
Sodium (mmol/L)	136–145	125	135		133
Potassium (mmol/L)	3.5–5.1	4.83	2.96		3.01
Chloride (mmol/L)	98–107	97.9	96.9		96.9
Creatinine (mg/dL)	0.7–1.2	0.41	0.36		0.39
Urea (mg/dL)	16.6–48.5	27.3	16.4		12.2
ALT (U/L)	0–41		14		13
AST (U/L)	0–40		26.1		15
ALP (U/L)	55–149		139		123
PICT	Nonreactive				
ESR (mm/hour)	< 20	110			
Serum albumin (g/dL)	3.5–5	1.18			
Total bilirubin (mg/dL)	0.3–1.2	0.959			
Direct bilirubin (mg/dL)	0.1–0.3	0.544			

Initial complete blood count showed leukocytosis of 32,000/mm^3^ with 95% neutrophils. ESR was elevated at 110 mm/hour. Sputum GeneXpert was positive for *Mycobacterium tuberculosis* and sensitive to rifampicin. PICT was nonreactive. Based on these findings, the diagnosis was revised to disseminated tuberculosis with severe pneumonia. Chest CT was performed, and anti-TB therapy was initiated: HRZE (isoniazid, rifampicin, pyrazinamide, ethambutol) four tablets daily for 2 months and RH for 4 months in accordance with the protocol along with pyridoxine 50 mg PO daily.

**Fig.1:**
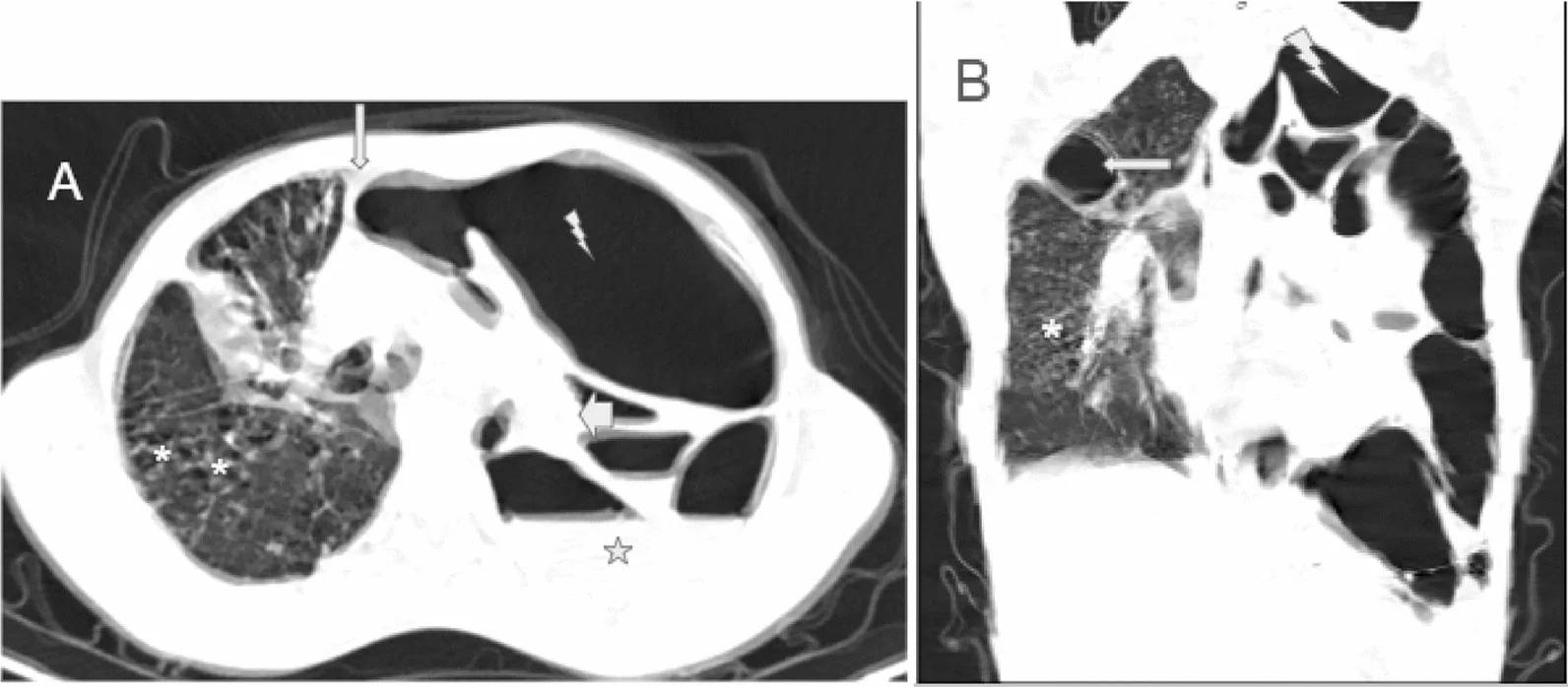
Initial noncontrast chest CT with HRCT image demonstrates left-sided tension pneumothorax (lightning bolt) evidenced by contralateral deviation of the anterior junctional line (down arrow) and mediastinum and collapsing of the lung parenchyma (short arrow) in image A. There is also a fluid-attenuating density (5 star) on image A, suggesting hydropneumothorax or pyopneumothorax. There are numerous tiny centrilobular nodules having a tree-in-bud pattern over the apical segment of the right lower lobe, with all segments of the RML and RUL (asterisks) making disseminated TB top differential. Image B shows a cystic dilatation of the bronchial lumen in the RUL. RUL: right upper lobe. RML: right middle lobe

**Fig. 2:**
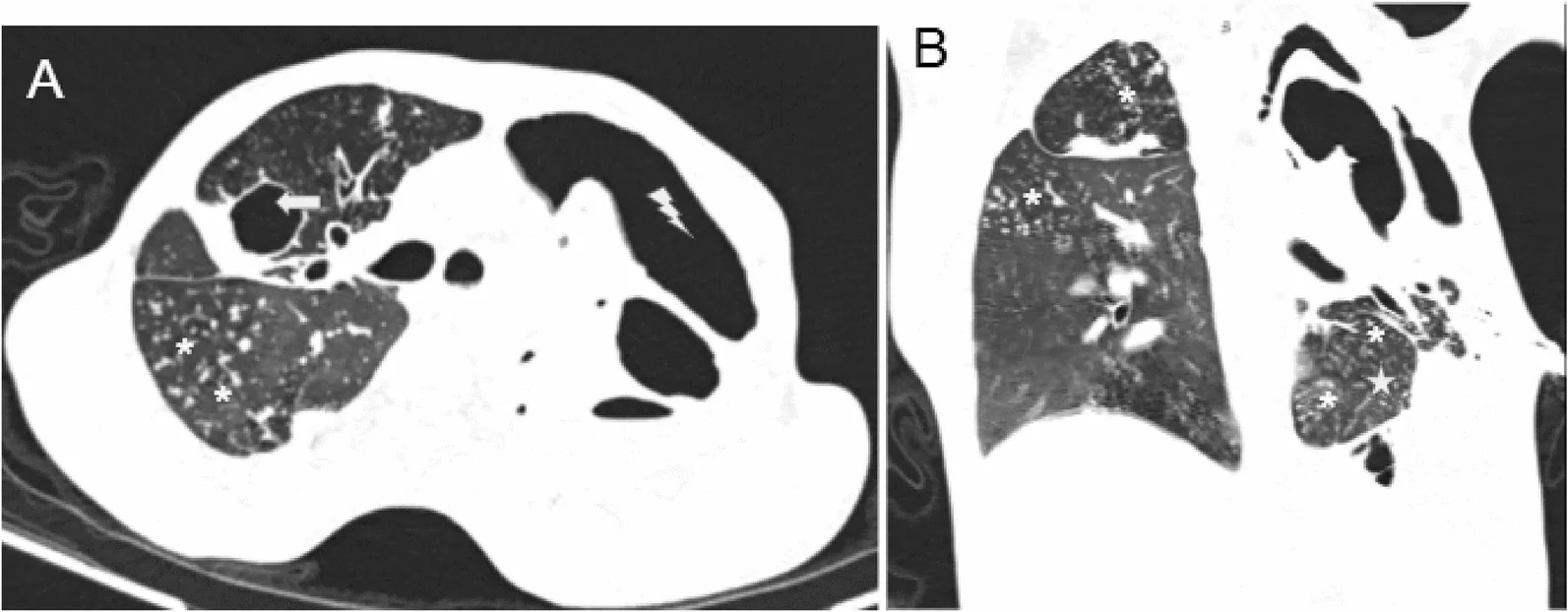
Follow-up noncontrast chest CT and HRCT after 10 days showing decreased pneumothorax evidenced by normal position of the anterior junctional line and partial expansion of the left lung. Chest tube is in situ

The initial chest CT (Fig. 1) revealed a left-sided tension hydropneumothorax, suggesting bronchopleural fistula. Numerous tiny centrilobular nodules with a tree-in-bud pattern were noted in the apical segment of the right lower lobe, and all segments of the right middle and upper lobes—findings most consistent with disseminated tuberculosis. Additionally, pleural fluid analysis showed a white blood cell count of 18,000/µL and 90% neutrophils (Table 1). Based on these findings, left-sided chest tube was inserted and the patient was transferred to the ICU, and the surgical team was consulted. Upon insertion, 200 mL of turbid fluid and a gush of air were observed. Plans were made to monitor chest tube output and bubbling. Following this, the patient’s oxygenation improved, but there was continuous bubbling from the underwater seal. Subsequently, the patient was transferred to the medical ward after a 5-day ICU stay where the same management was continued. A follow-up chest CT (Fig. 2), done 10 days after chest tube insertion, showed a reduction in pneumothorax, and partial re-expansion of the left lung, with the chest tube in situ.

**Fig. 3:**
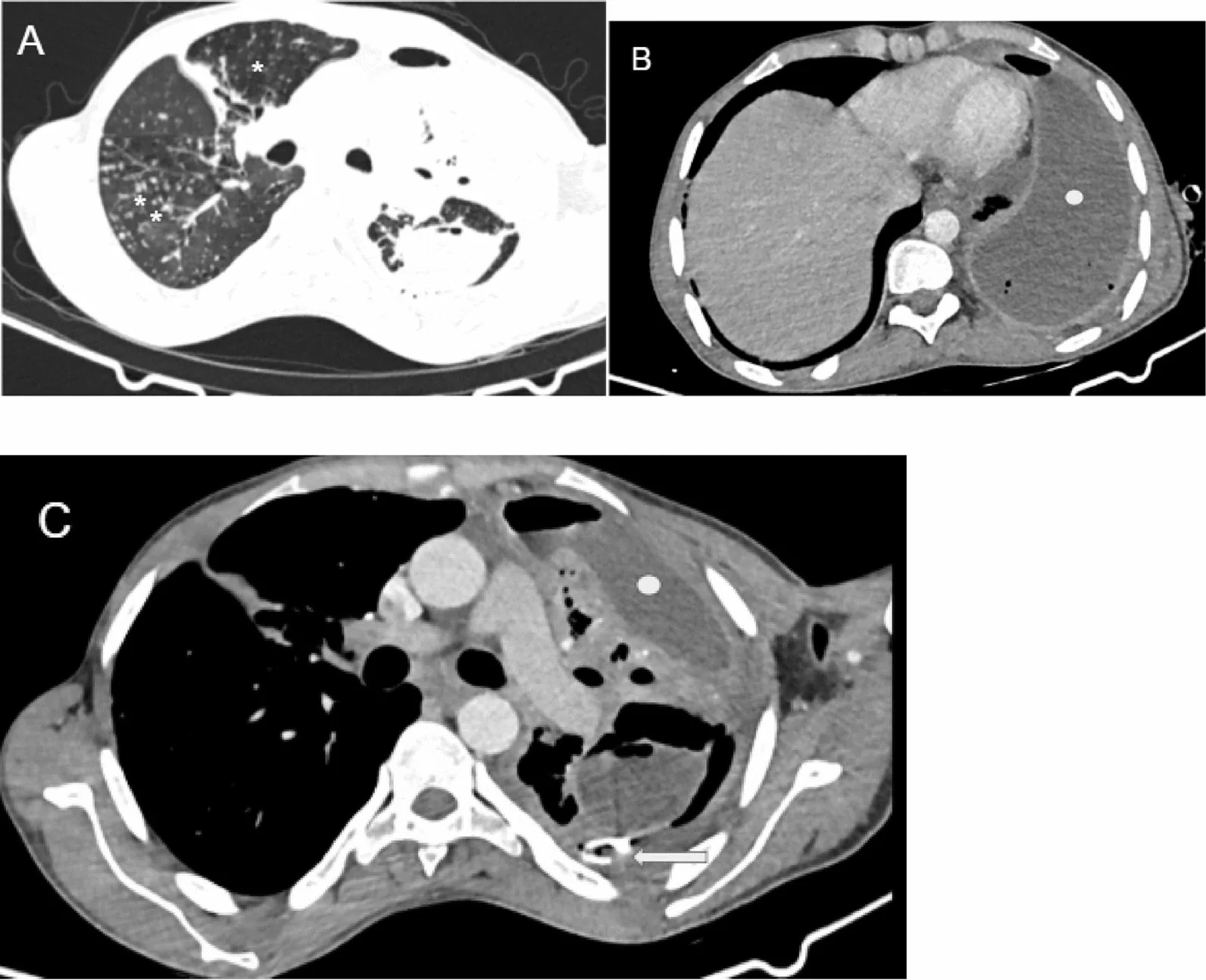
.Follow-up postcontrast chest CT after a month reveals multiloculated fluid-attenuating lesion with pleural split sign and air foci in the left pleural space suggesting an empyema. Chest tube is in situ

The treatment plan included continuation of antitubercular therapy, chest physiotherapy, and ongoing antibiotic therapy, with the addition of oral clindamycin 600 mg three times daily, along with close monitoring of chest tube output and pulmonary status. He took antibiotics for a total of 30 days. A follow-up chest CT performed 1 month after admission showed features suggestive of a left-sided pleural empyema (Fig. 3). He took antibiotics for a total of 30 days. The patient remained in the medical ward for 3 weeks, during which he showed progressive improvement in oxygenation and was weaned off supplemental oxygen. The chest tube remained in place for a total of 4 weeks and was removed once drainage and bubbling ceased and his clinical condition improved. Laboratory markers, including WBC count (Table 1), showed progressive improvement.

The patient was discharged on standard anti-TB therapy after a total hospital stay of 6 weeks. He was scheduled for follow-up at the infectious disease and pulmonology clinics. Unfortunately, he did not attend his first two follow-up appointments, and attempts to contact him via his personal phone were unsuccessful. A summary of the timeline of clinical events is provided in Table 2.

**Table 2:** Timeline of clinical events

Time point	Clinical events
Day 0 (presentation)	Patient presented with fever, cough, and dyspnea. CBC, pleural fluid analysis sent. Empirical intravenous antibiotics were initiated. Chest imaging showed tension hydropneumothorax. Chest tube was inserted
Day 1	Sputum GeneXpert resulted positive for *Mycobacterium tuberculosis*. Anti-TB therapy was initiated along with pyridoxine
Day 2	The patient was transferred to the ICU, and the surgical team was consulted due to continuous bubbling in the chest tube. A diagnosis of bronchopleural fistula was made, supported by the presence of a persistent air leak. Clinical monitoring was continued
Day 3–7	Ongoing ICU management included chest tube drainage, intravenous antibiotics, and close monitoring of respiratory status and chest tube output
Day 8–week 4	The patient was transferred to the medical ward, where the same management was continued. A follow-up chest CT demonstrated lung re-expansion, and clindamycin was added
Week 4–6	Gradual clinical improvement noted, with reduction in air leak and improved pulmonary status. Laboratory markers improved. Chest tube was removed. Follow-up chest CT was repeated
Week 6	The patient was discharged in stable condition on the initiation phase of antitubercular therapy, with plans for short-term follow-up

## Discussion

Tuberculosis (TB) is an infectious disease caused by the gram-positive, acid-fast bacillus *Mycobacterium tuberculosis*. While TB can affect almost any organ, the lungs remain the most commonly involved site [3]. Despite global public health efforts, TB continues to manifest with unusual and serious complications, particularly in young individuals, due to delayed diagnosis or incomplete treatment [3].

A bronchopleural fistula (BPF) is a pathological communication between the bronchial tree and the pleural cavity. It may result from surgical procedures, infections, blunt or penetrating chest trauma, radiation, chemotherapy, or as a sequela of chronic obstructive pulmonary disease [4]. In TB, BPF is an uncommon but severe complication with a wide spectrum of presentations[2]. The mechanism of tension physiology in tuberculosis is related to the formation of a bronchopleural fistula secondary to cavitary disease. In necrotizing pulmonary tuberculosis, cavitary lesions may erode into the pleural space and establish communication with the bronchial tree. Irregular tissue flaps at the fistula site, resulting from necrosis, inflammation, or scarring, can create a one-way valve effect, allowing air to enter the pleural space during inspiration while preventing its escape during expiration [9]). This leads to progressive intrapleural pressure buildup and may result in mediastinal shift and hemodynamic compromise.

Hydropneumothorax is a rare manifestation of tuberculosis and is more frequently associated with nontuberculous infections [3]. In TB, it may result from the rupture of a tuberculous abscess into the pleural space or obstruction and rupture of the thoracic duct [4]. In our case, the patient presented with left-sided tension hydropneumothorax, which makes this case particularly unique.

BPF poses significant diagnostic and therapeutic challenges. It is often diagnosed using a combination of clinical and imaging findings that confirm an air leak from the bronchial tree into the pleural space [5]. Patients usually present with respiratory symptoms such as cough, dyspnea, and fever, and, in some cases, with extrapulmonary complications. Erect chest radiography (revealing air–fluid levels) and computed tomography (CT) can be used to confirm the presence and extent of the fistula and rule out other causes [3]. This aligns with our case, where the patient exhibited classic symptoms of tuberculosis. Additionally, the chest CT revealed a left-sided bronchopleural fistula, along with hydropneumothorax, extensive parenchymal destruction, and multiple cavitations.

Differential diagnoses include a ruptured pyogenic lung abscess, fungal empyema (e.g., aspergillosis in immunocompromised patients), and malignancy-related fistulae. Microbiologic evaluation (GeneXpert and pleural fluid studies), along with serial imaging, helped confirm tuberculosis as the primary etiology. Secondary bacterial infection likely played a significant role, as evidenced by leukocytosis with neutrophilic predominance, necessitating broad-spectrum antibiotic coverage (initially cefepime and vancomycin, later including clindamycin). Although specific pleural fluid culture results were not available, the clinical picture suggests that secondary pathogens may have exacerbated parenchymal necrosis and fistula formation. Co-infection is common in advanced cavitary tuberculosis and is associated with poorer outcomes if not promptly addressed.

The management of bronchopleural fistula associated with tuberculosis is complex and requires a multidisciplinary approach, taking into account factors such as the patient’s clinical condition, infection status, and nutritional state. Anti-TB therapy forms the cornerstone of management[6, 7]8]. In select cases, intercostal drainage and suction provide sufficient control, while in others, surgical intervention becomes inevitable [9, 10].

Chest tube drainage is a fundamental component in managing tuberculosis complicated by pneumothorax or hydropneumothorax. The primary objectives are to drain pleural collections and facilitate lung re-expansion. However, challenges arise due to chronic inflammation, large fistulae, and delayed presentations, which can complicate the procedure[11]. This aligns with our patient, who presented with tension hydropneumothorax in the emergency department, leading to the insertion of a left-sided chest tube. However, despite the drainage of fluid, air bubbling was still observed in the underwater seal, indicating the presence of a bronchopleural fistula. The bubbling intensified when the patient coughed, confirming an air leak. Fortunately, the bubbling gradually diminished over the following weeks.

When medical therapy is ineffective, surgical interventions may be necessary. Options include open-window thoracostomy, decortication, thoracoplasty, or pleuropneumonectomy. Successful surgical outcomes depend on thorough preoperative planning, adequate antituberculosis therapy, and effective infection control [6, 10]. Conservative management was prioritized because of limited access to advanced thoracic surgical interventions (e.g., muscle flap closure, or open-window thoracostomy) in our setting, highlighting the diagnostic and management challenges in low-income countries. The patient’s young age, absence of major comorbidities, and rapid response to drainage and antimicrobials supported this nonoperative strategy.

In our review of cases, we identified two closely related instances. The first involved a 38-year-old male from the United Arab Emirates who presented with pneumothorax and hydrothorax. He was treated with a chest tube, antibiotics, and antitubercular therapy, and was discharged 23 days later. The second case was a 39-year-old male patient from Portugal, a smoker, who was diagnosed with a tuberculous bronchopleural fistula and unfortunately succumbed to refractory septic shock during his hospital stay. This patient also required single-lumen ventilation while on mechanical ventilation, but his underlying lung disease contributed to a poorer prognosis compared to the first case [3, 9].

Despite aggressive medical and surgical interventions, outcomes in severe TB-associated BPF can be poor, especially in patients with coexisting infections, severe malnutrition, or multiorgan dysfunction [1]. This case highlights the significance of early diagnosis, thorough supportive care, and effective interdisciplinary collaboration to enhance survival and successfully manage a tuberculous bronchopleural fistula through conservative means.

Limitations of this report include loss to follow-up after discharge, which precludes the assessment of long-term outcomes such as BPF recurrence, fibrothorax, or chronic respiratory impairment. Additionally, resource constraints limited advanced pleural fluid biochemistry (e.g., full Light’s criteria with protein/LDH/glucose) and microbiologic speciation. Despite these, the case demonstrates that timely intervention can yield favorable short-term results even in challenging environments.

## Conclusion

Bronchopleural fistulas (BPFs) are a rare and serious complication of tuberculosis, characterized by abnormal connections between the bronchus and the pleural space that permit air to enter the pleural cavity. The presence of hydropneumothorax complicates the condition further, making it even rarer. While surgical intervention may be necessary in some instances, conservative medical management is often a viable and preferred treatment option for tuberculous BPFs. Timely diagnosis and appropriate treatment are essential to prevent complications. A multidisciplinary approach is crucial to addressing the multisystem involvement and ensuring optimal care for patients with this serious complication of pulmonary tuberculosis. Close clinical and radiological follow-up is recommended for at least 6–12 months after discharge to monitor for BPF recurrence, residual air leaks, fibrothorax, or other long-term sequelae such as restrictive lung disease.

## Data Availability

Not applicable.
